# Diagnostic Utility of Immunohistochemical Expressions of IMP3 Versus DOG1 and p63 in Salivary Gland Tumors

**DOI:** 10.5146/tjpath.2020.01496

**Published:** 2020-09-15

**Authors:** Taiseer R. Ibrahim, Mona Mostafa Ahmed, Abdelmonem Awad Hegazy

**Affiliations:** Department of Pathology, Zagazig University, Faculty of Medicine, Zagazig, Egypt; Department of Anatomy and Embryology, Zagazig University, Faculty of Medicine, Zagazig, Egypt

**Keywords:** IMP3, DOG1, p63, Salivary gland tumors, Immunohistochemistry

## Abstract

***Objective:*** The diverse site of origin and classification complexity of salivary glands tumors increase difficulties in their diagnosis. This study aimed to evaluate the specificity and diagnostic ability of immunohistochemical expressions of IMP3 versus DOG1 and p63 in cases of such tumors.

***Material and Method:*** Thirty paraffin-embedded salivary gland tumors were obtained from the Pathology Department Archive. Their diagnosis was confirmed. The specimens were then re-classified and evaluated using the IMP3, DOG1 and p63 immunohistochemical markers.

***Results:*** There were 8 pleomorphic adenoma (PA), 12 mucoepidermoid carcinoma (MEC) and 10 adenoid cystic carcinoma (ADC) cases. All 12 MECs (100%) were IMP3 positive, while 30% of ADCs and only 25% of PAs were positive for IMP3. There was a statistically significant relationship between salivary gland tumors and IMP3 immunostaining (P =0.03). As regards to DOG1 results, 12.5% of PAs showed variable luminal positive immunostaining and 40% of ADCs showed weak luminal and abluminal immunostaining while 16.7% of MEC showed cytoplasmic staining. On the other hand, all ADCs (100%) showed moderate p63 reactivity in the nuclei of abluminal cells. All MEC cases (100%) were also p63-positive, showing a strong diffuse nuclear reactivity. A statistically significant relationship was noticed between salivary gland tumors and p63 immunostaining (P <0.05).

***Conclusion:*** IMP3 is more sensitive for diagnosis of MEC than ADC. p63 is statistically significant in diagnosing salivary gland tumors (MEC and ADC). On the other hand, DOG1 staining is not sensitive in diagnosis of studied malignant salivary gland tumors, limiting its diagnostic utility.

## INTRODUCTION

The salivary glands are exocrine organs consisting of ducto-acinar units. Their main function is the formation and secretion of saliva. There are major and minor glands. The major glands include three pairs: parotid, submandibular, and sublingual glands. The minor salivary glands are found in the oral cavity (1). The salivary glands have two basic types of cells: *luminal* that includes acinar and ductal cells, and *abluminal* in the form of myoepithelial and basal cells. The secretory acini and intercalated ducts are enveloped with myoepithelial cells, while the striated ducts and conducting portions are based on basal cells. Salivary gland tumors usually originate and differentiate along the same cell lines, i.e., epithelial (acinar and ductal), myoepithelial and basal. This could lead to a considerable overlap in diagnosis at all levels because all these cells can show various metaplastic changes such as oncocytic, sebaceous, squamous, and chondroid (2).

Salivary glands tumors represent about 1% of all neoplasms and 0.3% of human malignancies. Among head and neck cancers they represent 3% to 6% of cases. According to recent data, about 75% of salivary glands tumors develop in the parotid gland and only 20% of these are malignant. These tumors have extremely diverse morphology and heterogeneity with an unpredictable prognosis that complicate the therapeutic decision (3).

There are 34 different salivary gland epithelial tumors, including 10 benign and 24 malignant types, according to the 2005 third histologic classification of the World Health Organization. Common types of salivary gland tumors are mucoepidermoid carcinoma (MEC), pleomorphic adenoma (PA), adenoid cystic carcinoma (ADC) and Warthin tumor. Although the diagnosis of most salivary gland tumors can be made on the basis of hematoxylin and eosin-stained sections, immunohistochemistry can provide a powerful adjunct tool for pathologists to identify the cellular differentiation and assign correct classifications in difficult tumor cases (4).

The MEC represents the commonest primary malignant neoplasm in the salivary glands and accounts for 29 to 34% of these malignancies. This tumor has a unique cellular differentiation, being composed of a mixture of mucous, intermediate and squamoid cells, making the differential diagnosis of MEC broad (5). In addition, ADC is a malignant biphasic tumor composed of modified myoepithelial and ductal cells that commonly occur in the salivary glands, but may also be present in other organs like the lung, prostate, skin, and breast. ADCs are found in various patterns including cribriform, tubular and solid. Polymorphous low-grade adenocarcinoma may show a significant architectural and cytological overlap with ADC. In contrast to breast ADCs that have a favorable prognosis, the salivary gland counterpart shows poor long-term outcome (6). On the other hand, PA might be difficult to distinguish from several benign and malignant salivary gland tumors. Histologic findings that may represent diagnostic pitfalls include the presence of areas of squamous and mucinous metaplasia. This could mimic the appearance of MEC, or the presence of areas with cribriform architecture that may mimic the appearance of ADC (7).

The heterogeneity of cellular differentiation and the histological patterns makes the diagnosis of salivary gland tumors a challenging matter. The similarity in histological patterns among many different tumors further complicates the diagnosis with subsequent propensity for recurrence and metastasis. The histologic diversity of these tumors is due to the presence of myoepithelial cells; tumors containing myoepithelial cells exhibit slow progression and a low metastatic capacity. This requires investigating various biomarkers to support the histological diagnosis and to distinguish the different varieties of these tumors (8).

Insulin-like growth factor II m-RNA-binding protein 3 (IMP3) is an oncofetal protein. It belongs to the family of insulin-like growth factor II that is important in cell migration during early embryogenesis. It represents a component of RNA-binding protein required for early cleavage during pre-18s ribosomal RNA processing. IMP3 has been considered as a cancer-associated protein and its overexpression represents a prognostic marker in a variety of human types of malignancy (9).

The gene that encodes IMP3 is present on chromosome 7p15 and plays an important role in the migration and adhesion of cells in various malignant neoplasms. The 3 members of this family are known as IMP1, IMP2 and IMP3. IMP3 is strongly expressed in malignant tumors but rarely in normal adult tissues (8).

The gene known as anoctamin-1 (ANO-1), also known as discovered on GIST-1 (DOG1), was originally distinguished in gastrointestinal stromal tumors (GIST-1). Its function in secretory cell types such as those of the salivary gland is explained by its role in controlling the calcium-activated chloride channel; this explains its expression in salivary gland tumors indicating an acinic and intercalated duct cell origin (10).

p63 protein (p63) is a nuclear P53 homolog; it plays an essential role in the morphogenesis of the epidermis and limbs, in addition to acting as a transcription factor in the growth and development of many epithelial organs. It has been detected in basal stem cells of squamous epithelia as well as the basal cells/myoepithelial cells in the breast, sweat glands, prostate, and salivary glands (11).

The aim of this work was to evaluate the role and sensitivity of immunohistochemical expressions of IMP3 versus DOG1 and p63 (myoepithelial marker) in the diagnosis of salivary gland tumors.

## MATERIALS and METHODS

### Tissue Specimens

Thirty formalin-fixed and paraffin-embedded salivary gland tumors were randomly collected from the archive of Pathology Department, Faculty of Medicine, Zagazig University in the 2014 - 2017 period. We obtained the clinical and pathological information from the medical records of the patients. None of the patients had received chemotherapy or radiation therapy before surgery. Histopathological diagnosis and grading were done according to the World Health Organization classification (4). All paraffin blocks were cut at a thickness of 4 microns and the specimens were stained with the hematoxylin and eosin (H&E) stain to confirm the diagnosis.

The study was approved by the local ethics committee and patients’ consent were obtained.

### Immunohistochemical Procedures

Immunostaining was done using the standard avidin-biotin peroxidase method. Paraffin sections were de-paraffinized in xylene, and then rehydrated using descending grades of ethanol. Afterwards, antigen retrieval was done by treating the sections with 0.01 M citrate buffer (pH 6.0) for 30 minutes. After rapid rinsing in phosphate buffered saline (PBS), the sections were incubated in 0.3% hydrogen peroxide for 30 minutes to stop endogenous peroxidase enzyme (Dako ko411 kit). The sections were then treated with 5% horse serum for 2 hours at room temperature to inhibit the non-specific immunoreactions.

Primary monoclonal antibodies were incubated overnight in a humidity chamber (Table 1). After washing the sections in PBS, they were then incubated with biotinylated secondary antibodies for 30 minutes, and then treated with avidin-biotin peroxidase complex for another 30 minutes, according to the manufacturer’s instructions (Universal Detection Kit, Dako, Denmark). Finally, after visualization of the immune reaction using 3,3 - diaminobenzidine tetra hydrochloride (DAB, Dako K0114 Kit) for 5 minutes, the slides were counterstained with Mayer’s hematoxylin and mounted.

**Table 1:** Immunohistochemical markers.

**Antibody**	**Clone**	**Source**	**Dilution**	**Ag-retrieval**	**Positive control**	**Localization**
**IMP3**	Rabbit monoclonal antibodies	IMP3(clone; EPR 5111, Abcam, 1 Kendall Square, Suite B2304, Cambridge, MA02139-1517, USA)	1:50	Citrate buffer ph=6	Placental tissue	Nuclear and/ or cytoplasmic
**DOG1**	Rabbit monoclonal antibodies	DOG1 (clone 1.1, Thermo scientific catalog # MS-1933-P0)	1:50	Citrate buffer ph=6	Sections of gastrointestinal stromal tumor	Cell membrane and or cytoplasmic
**p63**	Rabbit monoclonal antibodies	p63 (Biocare medical, catalog # CM 163 B)	1:25	Citrate buffer ph=6	Nuclei of the basal epithelium in normal prostate	Nuclear

All the steps were performed at room temperature. Negative controls for all markers by omission of the primary antibody were performed. The surrounding normal salivary tissue was used as internal control.

### Interpretation of Immunohistochemical Markers

The immunostaining was semiquantitatively evaluated by 2 pathologists (TI &MM).

***Interpretation of IMP3 immunostaining*** **:** scoring was performed by counting the percentage of the positive cells: 0 if <10%; 1 if 10%-25%; 2 if 26%-50%; and 3 if >50%. Staining was seen in the nucleus or the cytoplasm or both. The reaction was considered positive if more than 10%; focal or heterogenous if more than 10% and less than 50%; and diffuse when more than 50% stained cells were present (5).

***Interpretation of DOG1 immunostaining:*** The staining intensity was graded as negative, weak (focal cytoplasmic staining), or strong (diffuse cytoplasmic staining). The reactions were considered positive if > 5% stained cells, further divided into focal if more than 5 and less than 10%, moderate between 10 and 50%, and diffuse if more than 50% of cells showed positivity (11).

***Interpretation of p63 immunostaining*** **:** Nuclear staining was scored as follows: Negative when <10% of nuclear stained cells; weakly positive from 10% to 25%; moderately positive from 26% to 75%; and strongly positive when >75% of nuclear stained cells (10).

### Statistical Analysis

The data were collected and analyzed using Microsoft Excel software, and then imported to the SPSS (Statistical Package for the Social Sciences) version 20.0 software for analysis. According to the type of data, qualitative variables were represented as numbers and percentages; and quantitative data represented by mean ± SD. The McNemar test and Kappa Agreement were used to investigate differences in variables for significance. The *P* value was considered significant if it was <0.05 and highly significant when <0.001. The validity of the immunohistochemical markers were measured by sensitivity, specificity, positive predictive value (PPV), negative predictive value (NPV) and diagnostic accuracy.

## RESULTS

The diagnosis of the thirty salivary gland tumors was PA in 8 and malignant tumors (12 MEC and 10 ADC) in 22.

### IMP3 Immunohistochemical Expression (Table 2, Table 3)

The surrounding normal salivary tissue beside the tumors was used as internal positive control. The ductal segment showed positive cytoplasmic IMP3 staining in luminal and extraluminal cells, while the intercalated duct showed cytoplasmic staining in the luminal cells only.

**Table 2:** Comparison between pleomorphic adenoma and malignant tumors as regard expression of immunohistochemical markers using the McNemar test.

			**Tumor type**		**Total**	**McNemar**	***P***	**Kappa agreement**
			**Pleomorphic Adenoma (n=8)**	**Malignant Salivary Tumors (n=22)**				
**IMP3**	**-ve**	**n**	6	7	13	2.75	0.045*	0.58
		**%**	75.0	31.8	43.3			
	**+ve**	**n**	2	15	17			
		**%**	25.0	68.2	56.7			
**DOG1**	**-ve**	**n**	7	16	23	1.71	0.39	0.109
		**%**	87.5	72.7	76.7			
	**+ve**	**n**	1	6	7			
		**%**	12.5	27.3	23.3			
**p63**	**-ve**	**n**	6	0	6	125.8	0.00**	0.83
		**%**	75.0	0.0	20.0			
	**+ve**	**n**	2	22	24			
		**%**	25.0	100.0	80.0			
**Total**		**n**	8	22	30			
		**%**	100.0	100.0	100.0			

**Table 3:** Comparison between malignant tumors as regard immunohistochemical markers using the McNemar test.

			**Tumor type**		**Total**	**McNemar**	***P***	**Kappa agreement**
			**MEC**	**ADC**				
**IMP3**	**-ve**	**n**	0	7	7	8.25	0.0002**	0.71
		**%**	0.0	70.0	31.8			
	**+ve**	**n**	12	3	15			
		**%**	100.0	30.0	68.2			
**DOG1**	**-ve**	**n**	10	6	16	1.82	0.291	0.32
		**%**	83.3	60.0	72.7			
	**+ve**	**n**	2	4	6			
		**%**	16.7	40.0	27.3			
**p63**	**+ve**	**n**	12	10	22	====	------	-----
		**%**	100.0	100.0	100.0			
**Total**		**n**	12	10	22			

**MEC:** Mucoepidermoid carcinoma, **ADC:** Adenoid cystic carcinoma.

**Figure 1:**
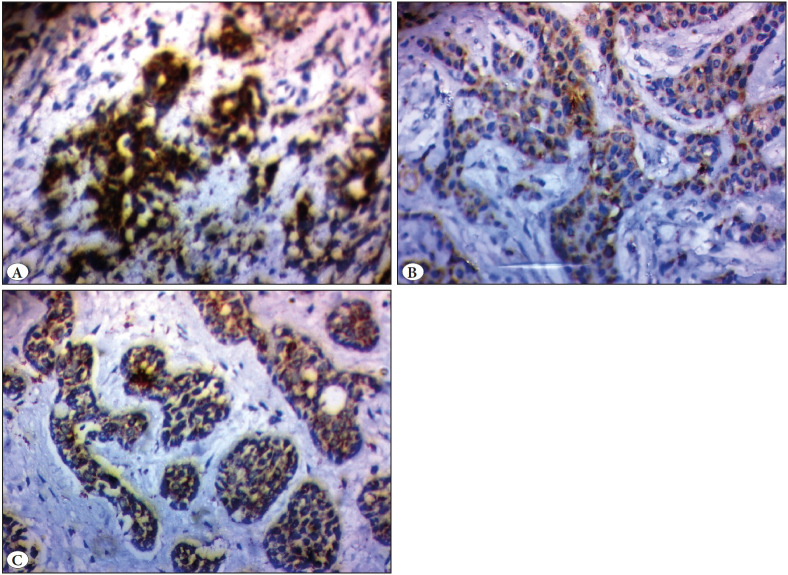
Representative samples of IMP3 expression in studied cases. **A)** Pleomorphic adenoma, score 3. (IHC; x200) **B)** Mucoepidermoid carcinoma with more than 90% of cells showing positive cytoplasmic expression, score 2. (IHC; x200) **C)** Adenoid cystic carcinoma with moderate positive cytoplasmic expression, score 2. (IHC; x200).

Only 2 cases of pleomorphic adenoma (25%) showed positive cytoplasmic immunostaining for IMP3 while 6 cases (75%) were negative. Staining was mainly observed in areas of squamous metaplasia (Figure 1).

All 12 MEC cases (100%) were IMP3 positive (Figure 1). Cytoplasmic granular staining was observed in areas corresponding to squamous and intermediate cells while mucosal cells were negative.

Three out of ten (30%) ADC cases showed positive IMP3 staining (Figure 1). In these tumors, cytoplasmic and membranous staining was detected in the cribriform and solid areas.

There was a statistically significant relationship between all cases of salivary gland tumors and IMP3 immunostaining (*P* =0.03).

### DOG1 Immunohistochemical Expression* *(Table 2, Table 3)

The normal salivary tissue adherent to tumors showed both membranous and cytoplasmic staining in serous acini at an apical/luminal side, while the intercalated ducts were focally positive and more proximal larger ducts showed negative staining.

**Figure 2:**
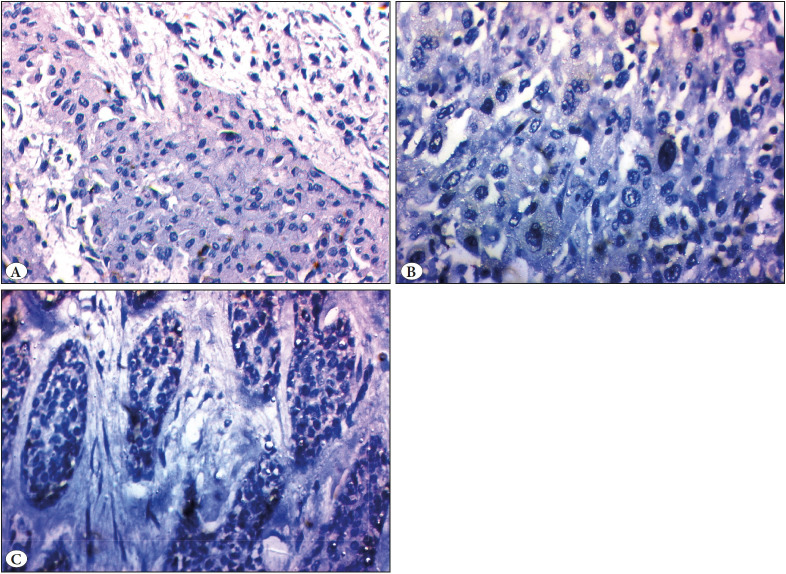
Immunohistochemical expression of DOG1 in studied cases. **A)** Mild expression in pleomorphic adenoma (IHC; x200). **B)** Negative expression in mucoepidermoid carcinoma (IHC; x400). **C)** Mild cytoplasmic immunostaining in adenoid cystic carcinoma (IHC; x200).

Only one case of PA showed variable luminal positive DOG1 (12.5%) (Figure 2).

Ten out of twelve MEC (83.3%) were DOG1 negative, while the remaining two cases (16.7%) showed weak positive cytoplasmic staining in the mucous and some of the intermediate cell components (Figure 2).

Six out of ten (60%) adenoid cystic carcinomas showed negative DOG1 staining while the remaining four cases (40%) showed weak luminal and abluminal immunostaining (Figure 2).

### p63 Immunohistochemical Expression* *(Table 2, Table 3)

p63 nuclear expression was found in the normal salivary tissue adjacent to tumors in the basal and myoepithelial cells.

**Figure 3:**
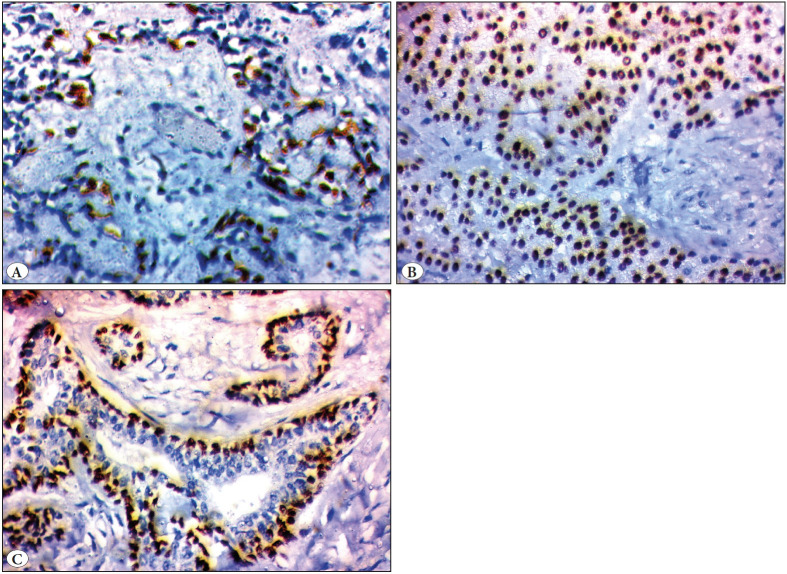
p63 expression in studied cases. **A)** Positive nuclear expression in myoepithelial cells in pleomorphic adenoma (IHC; x200). **B)** Mucoepidermoid carcinoma showing nuclear staining in squamous and intermediate cells only (IHC; x200). **C)** Nuclear immunostaining in abluminal cells while the luminal cells are negative in adenoid cystic carcinoma (IHC; x200).

Two out of eight cases of pleomorphic adenoma showed p63 staining in abluminal and myoepithelial cells (Figure 3).

All 12 (100%) MEC were p63 positive showing a strong diffuse nuclear reactivity in intermediate, squamous, and clear cells while the mucous cells were negative (Figure 3).

Moderate p63 nuclear reactivity was seen in all adenoid cystic carcinomas (100%) in the abluminal cells while it was absent in the luminal cells (Figure 3).

A statistically significant relationship was found obtained between salivary gland tumors and p63 immunostaining (*P* <0.05) (Table 2).

### Validity of Immunohistochemical Markers in the Diagnosis of the Studied Cases

In this study, we tested the validity of the markers in diagnosing studied cases. The sensitivity of IMP3 in diagnosing studied salivary gland tumors was 68.2%, the specificity 75%, the positive predictive value (PPV) 88.2%, negative predictive value (NPV) 46.1 % and diagnostic accuracy 70%. The sensitivity of DOG1 was 27.3 %, the specificity 87.5%, the PPV 85.7%, NPV 30.4%, and diagnostic accuracy 30.4%. Meanwhile, the sensitivity of p63 was 100%, the specificity 75%, PPV 91.6 %, NPV 100% and diagnostic accuracy 93.3% (Table 4).

**Table 4:** Diagnostic performance of immunohistochemical markers in diagnosis of studied cases.

	**Sensitivity**	**Specificity**	**+ve predictive**	**-ve predictive**	**Accuracy**
**IMP3**	68.2%	75%	88.2%	46.1%	70.0%
**DOG1**	27.3%	87.5%	85.7%	30.4%	30.4%
**p63**	100.0%	75%	91.6%	100.0%	93.3%

### Validity of IMP3 in the Diagnosis of the Studied MEC Cases

In this study, the sensitivity of IMP3 in diagnosing the studied MEC cases was 100%, the specificity 70%, the PPV 80%, NPV 100%, and diagnostic accuracy 86.3% For DOG1, the sensitivity was 40%, specificity 83.3%, PPV 66.7%, NPV 62.5%, and diagnostic accuracy 63.6% (Table 5).

**Table 5:** Diagnostic value of IMP3 and DOG1 in diagnosis of MEC.

	**Sensitivity**	**Specificity**	**+ve predictive**	**-ve predictive**	**Accuracy**
**IMP3**	100.0%	70.0%	80.0%	100.0%	86.3%
**DOG1**	40.0%	83.3%	66.7%	62.5%	63.6%

## DISCUSSION

IMP3 plays a vital role in cell migration in early embryogenesis; it is also required for ribosomal RNA processing. Expression of IMP3 is low or absent in adult tissues. The high and strong expression has been suggested to be a prognostic marker in a large variety of human types of cancers (8,9,12). In the present study, IMP3 expression was detected in the cytoplasm of normal salivary duct cells of adjacent normal tissues that was used as internal control. On the other hand, these findings were not detected in human breast and normal pancreatic tissue that did not show positivity for IMP3 but they are important in the differentiation between benign and malignant pancreatic lesions (12,13).

IMP3 expression was detected in 25% (2/8) of studied pleomorphic adenoma cases. These results differ from that of Isomerism et al. who reported that all cases of pleomorphic adenoma were positive for IMP3 (8). On the other hand, Elshafey et al. demonstrated that all cases of pleomorphic adenoma involved in their study and normal salivary gland tissues were negative for IMP3 staining (5). In studied cases of ADC, IMP3 expression was observed in 30% (3/10). Isomerism et al. found that IMP3 expression was present in 57% of cases of ADC (8).

In this study, all cases of MEC were positive for IMP3 expression. These findings are similar to that of Isomerism et al. (8). Elshafey et al. reported in their study that 51.4% of MEC cases were positive for IMP3 (5). In this study, the sensitivity of IMP3 in diagnosing studied MEC was 100%, the specificity 70%, PPV 80%, NPV 100%, and diagnostic accuracy 86.3%. Elshafey et al. reported that IMP3 expression is highly important in evaluating the outcome of MEC, and IMP3 can be used to differentiate MEC from PA (pleomorphic adenoma) of salivary glands (5). Isomerism et al. concluded that MEC seems to be more sensitive to IMP3 than ADC (8).

The difference was statistically significant between all studied tumors and between malignant ones (MEC and ADC) (*P* =0.03 and 0.0004 respectively). These findings seem to suggest that IMP3 staining differs between these tumors, allowing us to assume that this protein is an important biomarker of salivary tumors with squamous differentiation. Several studies have revealed the expression of IMP3 in squamous cell carcinoma which supports this hypothesis (14–17).

DOG1 is plasma membrane protein acting as a calcium-dependent chloride-channel and a marker of GIST (gastro-intestinal stromal tumor) by gene expression profiling. It is considered as a marker for differentiated acinic cells and intercalated duct cells as it is detected in secretory acini and diminished at the level of intercalated ducts and was completely absent more proximally in the normal salivary gland. This pattern of staining is in keeping with the secretory function of DOG1 (18–20). In the present study, only one case of pleomorphic adenoma (12.5%) showed DOG1 expression. These findings are similar to those of Khurram and Speight who reported that 28% of pleomorphic adenomas express DOG1 staining in the ductal component only (18). Andrade et al. demonstrated that DOG1 expression in benign salivary gland tumors like PA was similar to normal salivary gland tissues and concluded that it might be utilized as good marker for neoplastic cells derived from intercalated ducts or its progenitor cells (21). In this work, 83.3% (10/12) of MEC were negative for DOG1 and 16.7% (2/12) were positive. These results are similar to that of Abd Rabbah and Hakim who reported that 90.9% of MEC were negative for DOG1; and the positive cases (9.1%) stained both the mucous and some of the intermediate cell components (10). Also, Chenevert et al. found negative DOG1 staining in most mucoepidermoid carcinomas but the positive cases showed only focal weak staining in the mucous cell component (19). Khurram and Speight reported that MEC showed focal staining for DOG1 in 8 of 11 cases. In 3 of them, there was weak luminal staining and the remaining 5 showed weak or faint membranous expression in mucosal cells (18). In our study, four cases (40%) of ADC showed weak focal reactivity for DOG1. These results are similar to that of Khurram and Speight who reported that only two cases of ADC were positive for DOG1 expression (18). However, these are somewhat different to previous findings of Abd Rabbah and Hakim and Chenevert et al. who demonstrated DOG1 positivity in both ductal and myoepithelial components in 70-80% of cases and reported consistent luminal staining within the cribriform areas (10,19).

Yang et al. demonstrated in a recent study that DOG1(C kit) and p63 were expressed at rates of 61% and 64%, respectively, in salivary adenoid cystic carcinoma. They added that no significant differences in the expression of these markers among breast, salivary and metastatic ADC were noticed (6). The discrepancy between these studies might be due to the difference in antibody clones used in the different studies (18).

p63 is a marker for basal cells of the stratified epithelium and myoepithelial cells that occur to variable degrees in pleomorphic adenoma, adenoid cystic carcinoma and intermediate cells of mucoepidermoid carcinoma (22).

In the present study, only 25% (2/8) of pleomorphic adenoma showed positive p63 staining in abluminal and myoepithelial cells. On the other hand, Ladeji et al. demonstrated that all PA used in their study were positive for p63 antibody; and p63 staining was observed in the myoepithelial-like (abluminal) cells where they were seen to express a range of weak to moderate positivity (22).

Among all MEC cases, 12 (100%) were p63 positive with strong diffuse nuclear reactivity in intermediate, squamous and clear cells while mucous cells were negative. These findings are in line with Sams and Gnepp who demonstrated that salivary gland MEC showed positive staining for p63 in 100% of tumors in their study and added that p63 immunohistochemical staining can be useful in the differential diagnosis of acinic cell and MEC of the salivary, especially in mucin rich MEC, the mucous cells frequently will not stain with p63 while the adjacent basal and intermediate cells are always positive (23).

On the other hand, all adenoid cystic carcinomas (100%) showed moderate p63 reactivity in the nuclei of abluminal cells while the luminal cells were negative. These findings are consistent with other studies that reported that all adenoid cystic carcinomas showed positive moderate staining for p63 in abluminal cells only and strong positive nuclear staining for p63 in 100% of evaluated MEC (10,23–25). However, Khurram and Speight demonstrated that 75% of ADC showed positive p63 staining in abluminal and myoepithelial cells (18). In this study, a statistically significant relationship was found between salivary gland tumors and p63 immunostaining (P <0.05).

In this work, the diagnostic performance of the immunohistochemical markers was as follows: Sensitivity of IMP3 in diagnosing studied salivary gland tumors was 68.2%, the specificity 75%, the PPV 88.2%, NPV 46.1%, and diagnostic accuracy 70%. The sensitivity of DOG1 was 27.3%, the specificity 87.5%, the PPV 85.7%, NPV 30.4%, and diagnostic accuracy 30.4%. The sensitivity of p63 was 100%, the specificity 75%, the PPV 91.6%, NPV 100%, and diagnostic accuracy 93.3%. According to the current results, we suggest that p63 represents a reliable and statistically significant immunohistochemical marker in the diagnosis of salivary gland tumor. Butler et al. reported that the sensitivity, specificity, positive predictive value, and negative predictive value for p63 to detect MEC were 95.4, 87.2, 91.2 and 93.2%, respectively (26).

The limitations of this study include the low number of existing cases for each tumor type and absence of some tumor types due to their low incidence in our locality. Therefore, we recommend further studies for these markers on larger numbers of cases that involve all types of salivary carcinomas.

In conclusion, IMP3 has higher efficacy in diagnosis of mucoepidermoid carcinoma than adenoid cystic carcinoma, especially in problematic conflicts like suspicious cases or poor tissue sampling, and demonstrated the role of this protein in diagnosing salivary gland tumors. We think that IMP3 is a valuable marker of salivary tumors with squamous differentiation. DOG1 staining in myoepithelial cells is not reliable in diagnosis of mucoepidermoid carcinoma and adenoid cystic carcinoma, restricting its diagnostic utility. On the other hand, p63 is statistically significant in diagnosing salivary gland tumors (mucoepidermoid carcinomas and adenoid cystic carcinoma). Therefore, we suggest this panel of immunohistochemical markers in the diagnosis of challenging cases of salivary gland tumors.

## Conflict of Interest

The authors declare no conflict of interest.

## FUNDING

The authors declared that this study has received no financial support.
